# Phenology-Based Residual Trend Analysis of MODIS-NDVI Time Series for Assessing Human-Induced Land Degradation

**DOI:** 10.3390/s18113676

**Published:** 2018-10-29

**Authors:** Hao Chen, Xiangnan Liu, Chao Ding, Fang Huang

**Affiliations:** 1 School of Information Engineering, China University of Geosciences, Beijing 100083, China; haochen@cugb.edu.cn; 2 Key Laboratory of Digital Earth Science, Institute of Remote Sensing and Digital Earth, Chinese Academy of Sciences, Beijing 100094, China; dcgis2008@126.com; 3 School of Geographical Sciences, Northeast Normal University, Changchun 130024, China; huangf835@nenu.edu.cn

**Keywords:** land degradation, drylands, phenology, MODIS, NDVI time series, residual trend analysis

## Abstract

Land degradation is a widespread environmental issue and an important factor in limiting sustainability. In this study, we aimed to improve the accuracy of monitoring human-induced land degradation by using phenological signal detection and residual trend analysis (RESTREND). We proposed an improved model for assessing land degradation named phenology-based RESTREND (P-RESTREND). This method quantifies the influence of precipitation on normalized difference vegetation index (NDVI) variation by using the bivariate linear regression between NDVI and precipitation in pre-growing season and growing season. The performances of RESTREND and P-RESTREND for discriminating land degradation caused by climate and human activities were compared based on vegetation-precipitation relationship. The test area is in Western Songnen Plain, Northeast China. It is a typical region with a large area of degraded drylands. The MODIS 8-day composite reflectance product and daily precipitation data during 2000–2015 were used. Our results showed that P-RESTREND was more effective in distinguishing different drivers of land degradation than the RESTREND. Degraded areas in the Songnen grasslands can be effectively detected by P-RESTREND. Therefore, this modified model can be regarded as a practical method for assessing human-induced land degradation.

## 1. Introduction

Land degradation can be recognized as a continuing loss of ecosystem service due to loss of soil fertility, loss of vegetation cover and productivity, soil erosion, change in plant species, and other processes of environmental evolution [1]. The United Nations Convention to Combat Desertification (UNCCD) identified that land degradation has become one of the most pressing environmental problems in many countries [2]. The rate and extent of land degradation has both reached an astonishing level [3]. Many studies have indicated that human activities are the key drivers of land degradation [4,5]. For example, urbanization is one of the most widespread anthropogenic causes of land degradation in recent years [6,7]. Rapid changes in land use and land cover as well as the increased ecosystematic degradation were caused by the increasing rate of urbanization and increasing population in developing cities [8]. Considerable attention is currently being directed towards monitoring changes in the present state of degradation [9]. Such studies are important because the spatial characteristics of land degradation are useful for understanding the various impacts of human activities on the ecological condition. On the other hand, the health of human society is also influenced by land degradation. For example, approximately 2 billion people live in degraded regions [10]. Meanwhile, the low productivity in these regions shows the terrible impacts in politics and economics [11]. A target termed “zero net land degradation” was proposed by the UN Conference on Sustainable Development, and accurate quantification of land degradation has been designated as a high priority.

Assessing dryland degradation is problematic because dryland ecosystems generally go through high interannual variability in precipitation [12,13]. The climate variability results in great changes in interannual vegetation productivity, which affects the judgment of land degradation [14]. Field observations have been considered as an accurate means of identifying degradation for a long time [13,15]. An unfortunate reality is that the ground observation is scarce throughout the world because it is costly. Therefore, existing studies generally cover a small spatial scale [16], a short time span, or only measure parameters for usability in small landscapes rather than a whole ecosystem [17,18,19,20]. However, land degradation generally occurs progressively over a long time and encompasses a large area. Hence, remote sensing provides a unique opportunity for evaluating long-term land degradation over large areas.

Serious degradation generally lead to a long-lasting reduction in vegetation coverage and productivity [21]. Therefore, trend in vegetation productivity is regarded as an effective indicator of land degradation [22]. Vegetation indices based on reflectance of the near-infrared and visible spectra, such as the normalized difference vegetation index (NDVI), have been shown to be highly correlated with vegetation productivity [23,24]. Many studies on land degradation assessment are on the basis of the NDVI trend calculated from long-term satellite data [25,26,27,28,29,30].

Variation in the original NDVI cannot reflect human-induced land degradation directly because of the impact of precipitation. Hence, the major challenge to monitoring land degradation is to differentiate the impact of precipitation and human activities [31,32]. During the past several decades, many studies on this problem have been conducted. These studies can be divided into two major types: rain-use efficiency (RUE) and residual trend analysis (RESTREND). RUE is the quotient value of net primary production (NPP) to its corresponding precipitation [33]. Prince et al. (1998) investigated the RUE in Sahel from 1982–1994 and pointed out that decreased RUE referred to land degradation induced by reduced vegetation coverage and increased overland flow. Nevertheless, whether RUE is an effective indicator of land degradation is still disputable [34]. RESTREND was first proposed by Evans and Geerken in 2004. This method was based on a key hypothesis: NDVI is strongly correlated with precipitation in arid and semi-arid regions [35,36,37]. It has become the most widely used method to detect human-induced land degradation [38,39,40]. RESTREND first performs a linear regression between the accumulated NDVI and precipitation in the growing season. Next, the difference between the estimated NDVI value and the observed NDVI value is calculated, which is termed vegetation–precipitation relationship (VPR) residual. Finally, the VPR residual trend is estimated by using linear trend analysis. A negative trend in residual indicates land degradation [21,39]. RESTREND has been applied in many regions with different climate and landscapes [41,42,43].

Despite the good performance in land degradation monitoring, RESTREND has several limitations [21]. Particularly, the difference of vegetation growing season has not been paid sufficient attention. It is well known that vegetation growing season differs in time and location. However, previous studies have usually determined the growing season on the basis of priori knowledge [21,41], which is generally imprecise. On the other hand, the influence of precipitation before the growing season on NDVI is also not considered. This may bring in uncertainties because rainfall has lagging influence on vegetation growth. Vegetation indices time series have been widely used to determine the start and end of the growing season [44,45,46]. Using phenological information from vegetation indices time series is helpful to solve these limitations of RESTREND. 

In this study, we developed an improved RESTREND method by using phenological information to enhance the accuracy of detecting human-induced land degradation. A modified model named phenology-based RESTREND (P-RESTREND) was introduced here. We compared the P-RESTREND with RESTREND for detecting human-induced land degradation. Finally, we assessed land degradation/recovery in the Songnen grasslands using the P-RESTREND model.

## 2. Materials and Methods

### 2.1. Study Area

**Figure 1:**
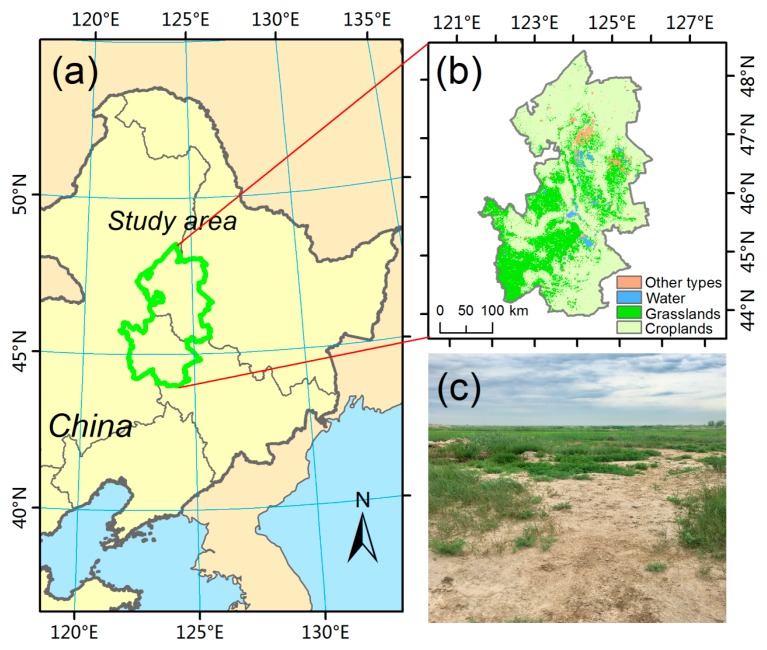
Geographical conditions of the study area. (**a**) Location of the Songnen grasslands; (**b**) land-cover types over the study area in 2013 from the MCD12Q1 product; (**c**) a photograph of the degraded grasslands in the study area.

We selected the Western Songnen Plain as the study area (Figure 1a). This area is under a semi-arid temperate continental monsoon climate. The main soil type is chernozem. The land-cover types are mainly grasslands and farmlands. The average annual precipitation ranges from 300 to 450 mm, of which over 70% is concentrated from June to September. We acquired the spatial distribution of grassland in the study area (Figure 1b) from MODIS land cover type product (MCD12Q1, with a 500 m spatial resolution). The available MODIS C5 MCD12Q1 data are from 2001 to 2013. The International Geosphere Biosphere Programme (IGBP) classification scheme was selected. Considering the influence of land cover change, only grasslands unchanged during 2001–2013 were retained. Large areas of grasslands are degraded because of unreasonable usage, restricting the sustainable development of this area. Figure 1c shows a photograph of the degraded grasslands in the study area.

### 2.2. Datasets

#### 2.2.1. MODIS Reflectance Data

The NDVI and NDWI time series were derived from the MODIS MOD09A1 surface reflectance product. MOD09A1 is an 8-day composite surface reflectance data, with a spatial resolution of 500 m. Each MOD09A1 pixel contains the best possible observation during an 8-day period as selected on the basis of high observational coverage, low view angle, absence of cloud, and aerosol loading. These data were already corrected for the influence of atmospheric gases and aerosols. We downloaded the Terra MOD09A1 data from 18 February 2000 to 30 December 2015 in the study area. NDVI and NDWI were calculated as follows:

(1) $$\mathrm{NDWI}=\frac{\left(\rho_{b2}-\rho_{b6}\right)}{\left(\rho_{b2}+\rho_{b6}\right)}$$

(2) $$\mathrm{NDVI}=\frac{\left(\rho_{b1}-\rho_{b2}\right)}{\left(\rho_{b1}+\rho_{b2}\right)}$$

where $\rho_{b1}$, $\rho_{b2}$, and $\rho_{b6}$ represent the reflectance of Band 1 (620–670 nm), Band 2 (841–876 nm), and Band 6 (1628–1652 nm) of MODIS data, respectively. In fact, MODIS also includes another two bands of water absorption: Band 5 and Band 7. Band 6 is the most commonly used band for calculating NDWI from MODIS.

#### 2.2.2. Precipitation Data

The daily precipitation data with 0.5° spatial resolution from 2000 to 2015 were downloaded from the Meteorological Data Center of the China Meteorological Administration (http://data.cma.cn/). These data were produced from thin plate spline (TPS) by using digital elevation model (DEM) data (GTOPO30) and relevant meteorological data. We converted the daily precipitation data to a 10-day temporal resolution. Considering the difference in spatial resolution between MODIS vegetation indices and the precipitation data, we resampled the precipitation data to a 500 m spatial resolution.

### 2.3. Methods

**Figure 2:**
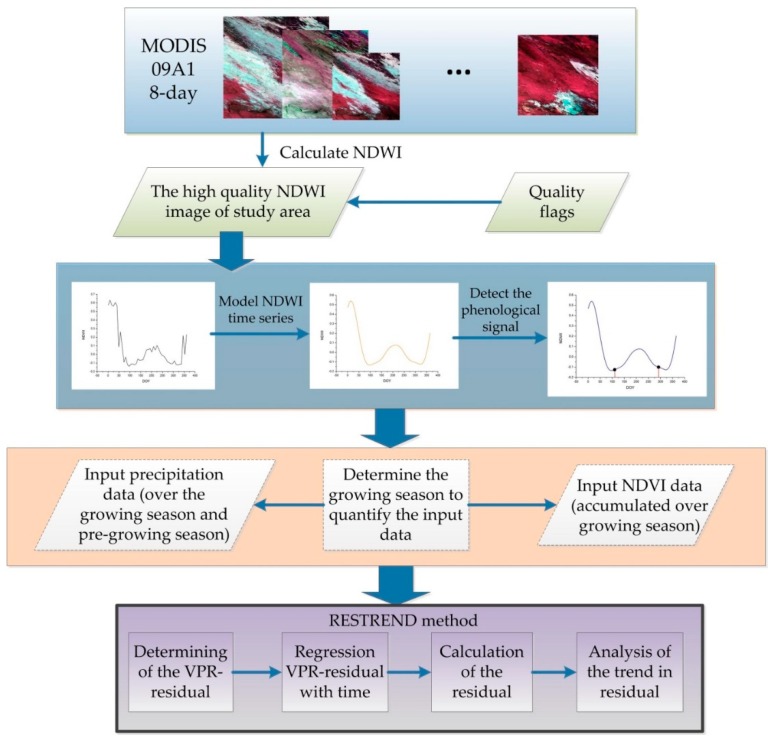
Flowchart of the P-RESTREND model.

Figure 2 shows the framework of the proposed method. The method mainly includes two parts: (1) quantification of the relationship between precipitation and NDVI by using phenological information; (2) analysis of the trend in VPR residual for detecting human-induced land degradation.

#### 2.3.1. Extraction of Growing Season Using NDWI Time Series

**Figure 3:**
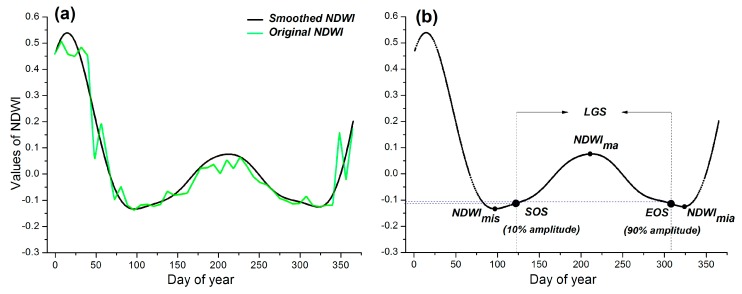
(**a**) Original and smoothed NDWI time series; (**b**) an example of detecting phenology from NDWI time series.

The curve of NDVI can be affected by the seasonal snow cover (generally from November to April) in the study area. On the other hand, vegetation growth status can be characterized by vegetation water content. Therefore, we selected NDWI time series to detect phenological information in our model. NDWI, which was proposed by Gao (1996), was based on near infrared (NIR) and short-wave infrared (SWIR) bands. It is an effective index for detecting vegetation water content [47]. In addition, the NDWI was demonstrated to be more efficient in distinguishing snowmelt from vegetation growth. For example, it is more suitable for detecting the phenological information than NDVI, which is seriously affected by snow cover in boreal regions [48]. We reconstructed the NDWI time series by using stationary wavelet transform. The quality flag in MOD09A1 was used to reduce the influences of cloud and cloud shadow. If the flag of an observation in the NDWI time series was cloud or cloud shadow, we replaced the corresponding NDWI value by linear interpolation from the nearest observations. We then performed the stationary wavelet transform to smooth the time series. The decomposition level was three, and the mother wavelet was Daubechies 3. The two highest frequency signals were regarded as noise [49]. We obtained the daily NDWI time series using cubic spline interpolation after the aforementioned processes (Figure 3a).

Considering the growth of vegetation in semi-arid ecosystem is closely related to the rainfall, the thresholds method was adopted for detecting phenology in this research. The minimum value during spring (NDWI_mis_), the minimum value during autumn (NDWI_mia_), and the maximum value (NDWI_ma_) between NDWI_mis_ and NDWI_mia_ were detected first. The NDWI_mis_ and NDWI_ma_ values were used to determine the amplitude of spring growth process. The NDWI_ma_ and NDWI_mis_ were used to determine the amplitude of autumn decay process in NDWI time series. The timing of SOS (start of the growing season) was defined as follows:

(3) $$T_{\mathrm{SOS}}=\max\left(\left(t\epsilon \left[{\mathrm{NDWI}}_{\mathrm{mis}},{\mathrm{NDWI}}_{\mathrm{ma}}\right]\right)\;|\;\left({\mathrm{NDWI}}_{\left(t\right)}<{\mathrm{NDWI}}_{\mathrm{mis}}+\varepsilon \right)\right)$$

where ε is the threshold, and T_SOS_ is the timing of SOS. We defined the date that equals to 10% amplitude of the spring growth process in the modeled NDWI time series as SOS. It has been widely used for the detection of phenology [50,51]. The EOS (end of the growing season) was detected by using the 10% amplitude between NDWI_ma_ and NDWI_mia_ (Figure 3b). 

#### 2.3.2. Phenology-Based RESTREND (P-RESTREND) for Assessing Land Degradation 

**Figure 4:**
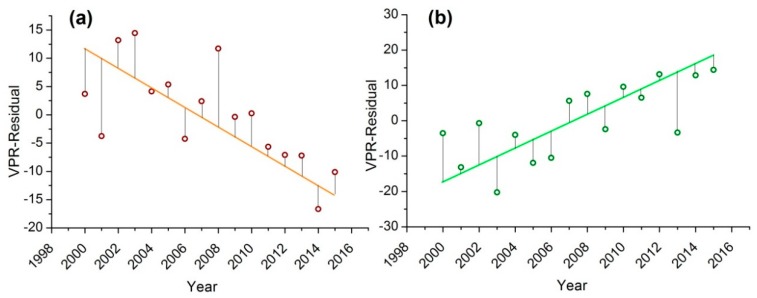
Ordinary least-squares (OLS) linear regression between the vegetation-precipitation relationship (VPR) residual and time: (**a**) a negative trend and (**b**) a positive trend in VPR residual.

Accumulated NDVI over the growing season (NDVI_acc_) is a typical indicator of vegetation productivity [29,52,53]. P-RESTREND determined land degradation by analyzing the variation in NDVI. As a pixel-based method, we first modeled the NDVI time series by using the same process as NDWI, and calculated NDVI_acc_ and accumulated precipitation over the corresponding growing season. P-RESTREND is based on a hypothesis: the change in NDVI is not only influenced by the precipitation over the growing season, but also influenced by the precipitation before SOS. We used the precipitation occurred over the month before SOS (hereafter known as precipitation over pre-growing season); a binary linear regression was used to evaluate the vegetation–precipitation relationship (VPR):

(4) $${\mathrm{NDVI}}_{\mathrm{accp}}={\alpha P}_{\mathrm{green}}+{\beta P}_{\mathrm{pgreen}}+\gamma$$

where NDVI_accp_ is the predicted NDVI_acc_; P_green_ and P_pgreen_ is the accumulated precipitation over the growing season and pre-growing season, respectively; α and β are the weight of P_green_ and P_pgreen_, respectively, and γ is the intercept. The difference between the observed NDVI and the NDVI estimated from VPR was referred to as the VPR residual. The residual trend was then calculated by ordinary least-squares (OLS) linear regression between the VPR residual and time, as shown in Figure 4. The trend in VPR residual can be regarded as independent to precipitation [39]. We then evaluated the significance of residual trend by hypothesis testing, and the significant trend indicates the process of land degradation or recovery.

#### 2.3.3. Comparison of RESTREND and P-RESTREND

VPR was used to assess the performances of RESTREND and P-RESTREND in differentiating precipitation- and human-induced land degradation. Three trials of the VPR test were conducted as follows:

1. The standard RESTREND was performed. Considering the geographical and climatic conditions of the study area, we determined the period May–September as grassland growing season.
2. Considering the different phenological behaviors of different pixels, we first extracted the growing season by the thresholds method and then performed the RESTREND method according to different phenological information pixel by pixel.
3. The P-RESTREND method was performed.

We then compared the significance of the VPR of the three trials to verify the performance of P-RESTREND in removing of the precipitation influence on NDVI.

In addition, the degraded areas in Songnen grassland were detected by RESTREND and P-RESTREND. The pixel that presents a significant land degradation trend in P-RESTREND but did not show significant degradation in the standard RESTREND was extracted. These pixels are hereafter known as missed pixels. We used the high-resolution remote sensing images obtained from Google earth to observe land surface changes by artificial interpretation. We selected three images for a missed pixel with a time interval of five years starting from 2000. If the image is missing in a specific year, we chose the nearest year to replace it. If the missed pixels are interpreted as degraded areas, it suggests that the P-RESTREND was more effective than the standard RESTREND.

## 3. Results

### 3.1. The Difference of Grassland Phenology across the Study Area

**Figure 5:**
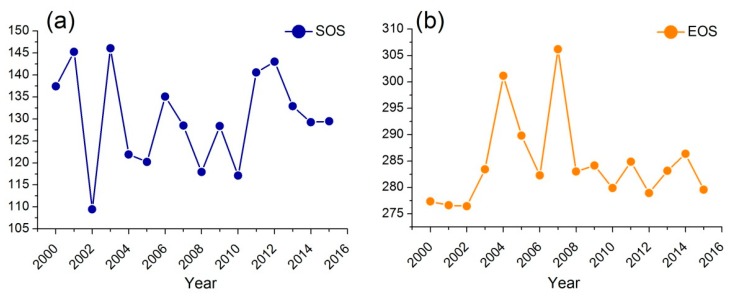
Mean values of interannual variability in (**a**) the start of the growing season (SOS) and (**b**) the end of the growing season (EOS) in the whole study area.

Approximately 30,000 pixels from MODIS data were analyzed in the study area to test the model. We averaged the pixel information over the study area and analyzed the interannual variability in SOS and EOS. As shown in Figure 5a, the fluctuation of SOS is significant and erratic. In particular, the biggest difference of SOS is over 30 days between 2002 and 2004. Simultaneously, the EOS also shows huge differences among years (Figure 5b).

**Figure 6:**
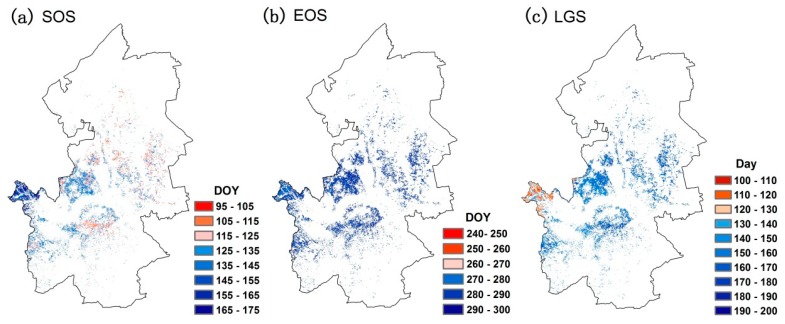
The spatial distributions of the multiyear mean value of (**a**) the SOS, (**b**) the EOS, and (**c**) the length of the green season.

Generally, for the mean date of SOS and EOS during 2000–2015, different regions show great differences. Spatial distributions of the mean dates of SOS and EOS are shown in Figure 6. An earlier SOS often occurs in the north-central regions rather than other regions in the study area (Figure 6a). The central region in the study area often presents a later EOS. Regarding the mean length of the green season (LGS), we found that the LGS in the western grassland is much shorter than that in other regions. These results indicate the necessity of using phenological information to define the growing season.

Though the field observation data is lacking in the Songnen grassland, a number of studies exists that can be compared to the results of this study [54,55]. The results of this study showed that the mean SOS was primarily between DOY 105 to 150. The EOS in our study was mainly between DOY 275 to 310. By comparison, we found that the phenological information we extracted was similar to existing studies. For example, Liu et al. showed that the average EOS was mainly from DOY 270 to 310 [54]; Zhao et al. showed that the mean SOS was mainly from DOY 110 to 150 [55].

### 3.2. Comparison of VPR Determined by Three Trials

Pixel information was averaged to compare the VPR. Table 1 suggests that P-RESTREND had better performance in differentiating precipitation and human-induced drivers of land degradation, with the mean value of R^2^ reaching 0.45. It met our expectations, and we also found that the second trial’s R^2^ (0.35) is slightly lower than the first trial’s R^2^ (0.38) in Table 1. 

**Figure 7:**
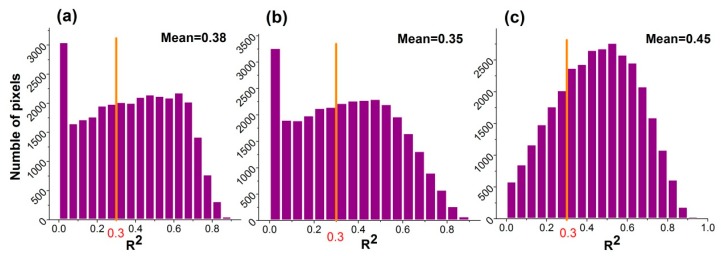
Statistics of R^2^ between NDVI_acc_ and precipitation of (**a**) RESTREND, (**b**) the second trial, and (**c**) P-RESTREND.

RESTREND has a criterion of application on a pixel: the VPR should be significant and strong. Wessels et al. (2012) suggest a value of R^2^ > 0.3 [21]. The frequency distributions of R^2^ are shown in Figure 7. As expected, approximately 75% of the pixels met the criteria using the P-RESTREND, which was the highest proportion among the three trials. The proportions of pixels meeting the criteria of the RESTREND and the second trial were 61% and 58%, respectively.

**Figure 8:**
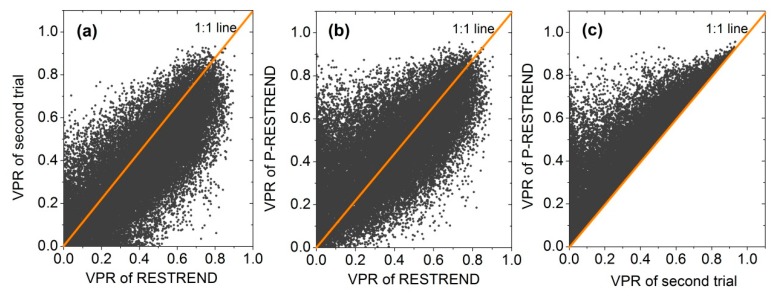
Pixel-by-pixel comparison of R^2^ between (**a**) RESTREND and the second method, (**b**) RESTREND and P-RESTREND, and (**c**) the second method and P-RESTREND.

In addition, we compared the VPR based on each individual pixel for three trials. The performance of P-RESTREND was also better than RESTREND (Figure 8). For most pixels, the R^2^ of P-RESTREND was higher than that of the other two trials (Figure 8b,c). The significance of VPR is similar between RESTREND and the second trial, which can also be observed in Figure 8a. The results indicated that the P-RESTREND, compared to the standard RESTREND method, had greater accuracy in discrimination of land degradation caused by climate and human activities.

### 3.3. Land Degradation in Songnen Grasslands Detected by Different Methods

#### 3.3.1. Land Degradation Detected by P-RESTREND and RESTREND

**Figure 9:**
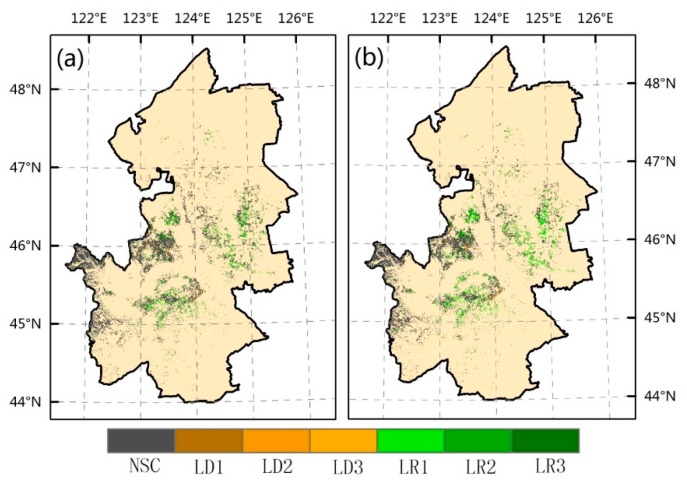
The trend and significance of VPR residual detected using (**a**) P-RESTREND and (**b**) RESTREND. The significance classes for land recovery are as follows: LR1 (P < 0.01), LR2 (0.01 < P < 0.05), and LR3 (0.05 < P < 0.1). The significance classes for land degradation are as follows: LD1 (P < 0.01), LD2 (0.01 < P < 0.05), and LD3 (0.05 < P < 0.1). No significant change is NSC (P > 0.1).

We categorized the pixels into seven classes by calculating the trend of the VPR residual and its significance. For the regions where the productivity increased (presenting land recovery) LR1 (P < 0.01), LR2 (0.01< P < 0.05), and LR3 (0.05 < P < 0.1); for the areas where the productivity decreased (presenting land degradation) LD1 (P < 0.01), LD2 (0.01 < P < 0.05), and LD3 (0.05 < P < 0.1). We defined the last category as no significant changes in productivity NSC (∝ > 0.1). Figure 9 shows the significance and direction in grassland productivity in Songnen Plain between 2000 and 2015 by using P-RESTREND and RESTREND. According to statistics, a total of 29.81% pixels showed a significant trend with 2.44% were negative and 27.37% were positive when we adopted P-RESTREND in the study area; similarly, there are 1.89% of the pixels showed the significant land degradation trend, and 25.68% of the pixels showed the significant land recovery trend while using the RESTREND method (Table 2). Most pixels that presented no significant changes in productivity can be observed in both methods. 

As shown in Figure 9, no matter which method we adopt, positive trends are widespread throughout the study area, particularly apparent in the eastern grassland and southern grassland. Negative changes are generally concentrated in some areas near the latitude of 46 degrees, in the central of our study area. In addition, the grassland located in southeastern area of the study area had small-scale degradation, which can be observed in Figure 9.

#### 3.3.2. Changes of Land Surface in “Missed Pixels” by Artificial Interpretation

**Figure 10:**
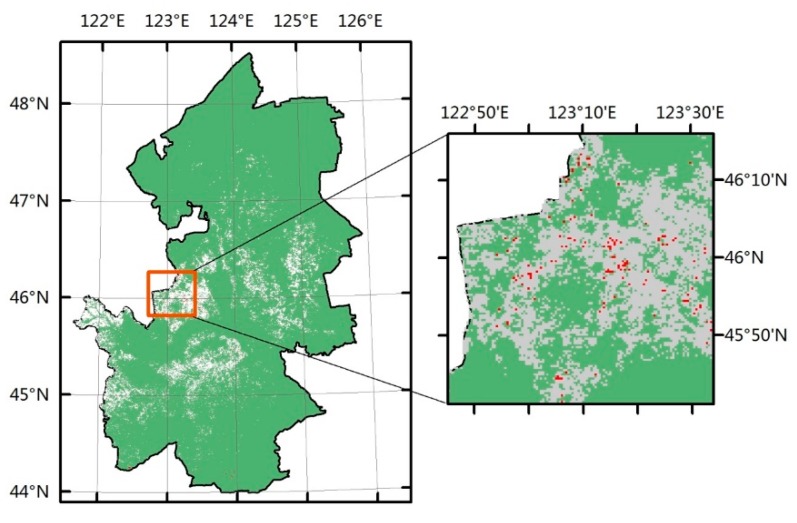
The spatial distribution of significant degradation pixels detected by P-RESTREND but was not detected by RESTREND.

Both methods, though, show similar trends of land degradation in the study area. There were approximately 0.6% of the pixels are missed pixels. Figure 10 showed the spatial distribution of the missed pixels. In general, the distribution of these areas is scattered throughout the study area and mainly centralized in the central and eastern of the study area, which is shown in Figure 10.

**Figure 11:**
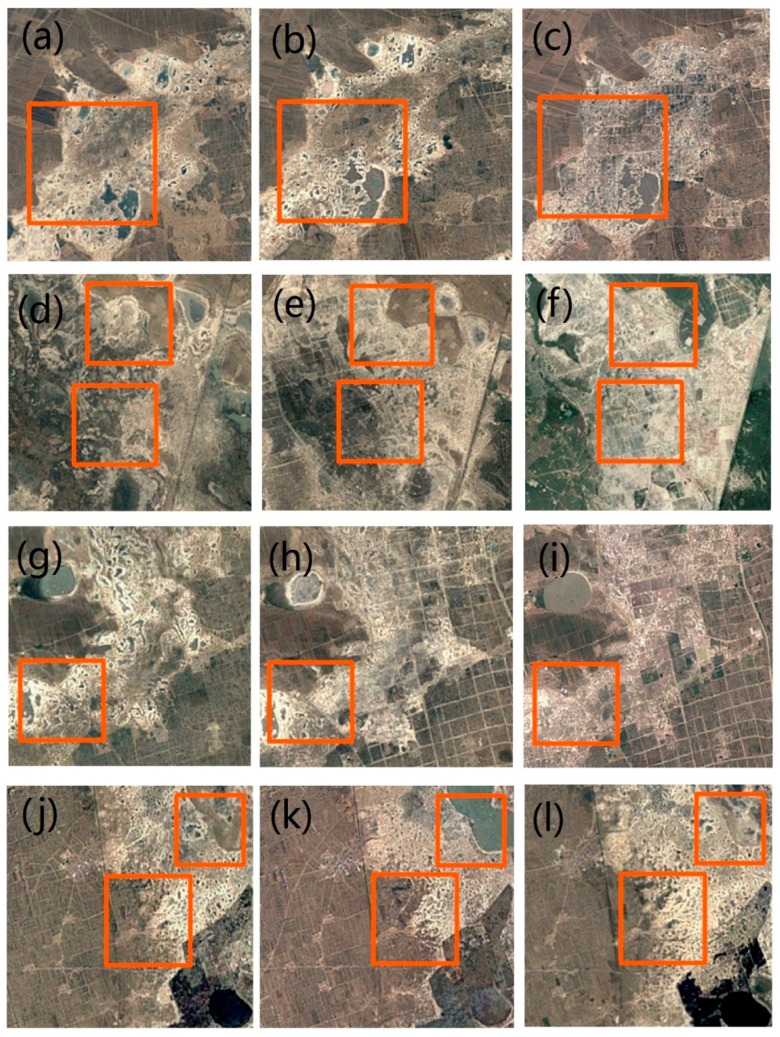
The high-resolution remote sensing images ((**a**,**d**,**g**,**j**) obtained in 2000–2004; (**b**,**e**,**h**,**k**) obtained in 2005–2009; (**c**,**f**,**i**,**l**) obtained in 2010–2015) of four different missed pixels (the images of different pixels was placed in different rows). The areas with significant changes are marked by the orange rectangle.

Because the missed pixels are scattered throughout the study area, the remote sensing images for some pixels are missing. On the other hand, the land degradation is not serious in some missed pixels; we could not observe the clear signs of land degradation. Therefore, we selected approximately 40% of the missed pixels where high-resolution remote sensing images were available to observe the changes of land surface. The result showed that the areas of desertification obviously increased in most of the missed pixels, which is the typical form of land degradation. In particular, most missed pixels showed obvious signs of human activity. By artificial interpretation, we found that P-RESTREND was more effective in monitoring land degradation than RESTREND, because these missed pixels have the sign of land degradation indeed. We randomly selected some pixels among the missed pixels to show these typical changes in Figure 11.

## 4. Discussion

The climate influence on vegetation productivity trends seriously interferes with the monitoring human-induced land degradation by remote sensing data [31,32]. The purpose of this study was to more effectively remove the influence of precipitation on the interannual changes in NDVI that can improve the accuracy of monitoring land degradation. To achieve this goal, we considered the influence of precipitation before the growing season on NDVI and the difference of growing season across a large region. The results of the modified methodology named P-RESTREND were compared with the typical RESTREND. Notably, P-RESTREND can distinguish different drivers of land degradation more effectively than RESTREND can. Furthermore, we detected the degradation areas in Songnen grassland based on RESTREND and P-RESTREND, and extracted the missed pixels mentioned above. The result of artificial interpretation showed that many of these missed pixels were land degradation pixels, which cannot be detected by RESTREND. Therefore, the modified method has better applicability than RESTREND for large-scale studies. 

The comparison of VPR between the standard RESTREND and the second trial as described above presented a strange result: RESTREND had a slightly better performance in removing the precipitation influence on vegetation productivity changes instead of the second trial, which we speculated was caused by the long and cold winters in Songnen grassland. The SOS is concentrated in May, while in some cold regions, the SOS occurs even later in the year. In this study, the growing season was assumed as the time from May to September when we performed the standard RESTREND. It is very possible that a part of the precipitation over the pre-growing season was covered in the VPR calculation. This assumption, which may make the VPR was more significant than the second trial. We also speculated that the precipitation over the growing season was not the only driver of NDVI changes based on this result. In particular, as shown in Figure 8b, the performance of P-RESTREND in removing the precipitation influence was not better than that of RESTREND for all pixels. It may relate with the low utilization of precipitation before the SOS for some regions. There are many reasons for this: increasing in surface runoff caused by rapid land degradation, temperature abnormalities before the SOS resulting in high evaporation, abnormal precipitation, etc. All of these factors can result in a relatively low R^2^, because the rainfall over the pre-growing season can be ignored in these conditions. On the other hand, the response between precipitation and the NDVI is complicated. Although precipitation over the pre-growing season has correlation with NDVI to some extent, this linear relationship is not completely uniform for each time period. This is related to the different growth conditions of vegetation at each period. The timing of rainfall is closely related to vegetation productivity, but hard to quantify. That is to say, correlation between precipitation and NDVI is perhaps the highest during May–September in several regions, even if we consider precipitation over the pre-growing season.

The improved performance of the modified methodology highlights the importance of considering different phenological signals and precipitation periods for each pixel. The modified algorithm presented in this study has several desirable properties. RESTREND assumes that the growing season is similar in temporal-space, which is unreasonable in several situations. In fact, the temporal-spatial differences of phenological information are huge, which has been observed in Figure 5 and Figure 6. Moreover, precipitation during the pre-growing season was also ignored in the RESTREND method. Our modified methodology comprehensively considered the phenology of grassland. We also characterized the influence of precipitation on NDVI by different weights. This operation partly eliminates the irrationality of RESTREND, which cannot accurately detect the degradation pixels in our study area. This suggests that, if the influence of precipitation has not been removed accurately, the residual trends will not veritably reflect the changes in vegetation productivity caused by human-induced land degradation [39]. Therefore, the results of RESTREND we obtained in this study were not satisfactory. Simultaneously, the remote sensing images with high spatial resolution were used to verify the situation of land degradation in missed pixels. The results showed that these areas had significant signs of human activities and the loss of grassy areas, which further confirms the validity of the modified methodology. Notably, the verification method was designed for missed pixels; it is only applied to small areas that like a single pixel. That is to say, the verification method cannot be regarded as an effective tool for land degradation monitoring on a region scale.

Some limitations also exist in our study. P-RESTREND was based on phenological signal detection, and any false extraction of growing season will exert an adverse impact on the entire technique, for which the time series should be reasonably modeled and practical method for phenology detection should be selected. Moreover, this study used the linear model to characterize the VPR, the nonlinear response between precipitation and NDVI was not considered in our methodology. Finally, an arid ecosystem which occurred major structural changes result in an abnormal VPR; the P-RESTREND also does not perform well. We did not take these abnormal changes into consideration, which is one of our directions of further work.

## 5. Conclusions

This research demonstrated the feasibility of improving the RESTREND by introducing phenological information to remove the influence of precipitation on NDVI for detecting human-induced land degradation. We used the NDWI time series to detect the growing season in regions with seasonal snow cover. We then developed a modified method to estimate vegetation–precipitation relationship using phenological information. We tested the proposed method through comparison of different trials and demonstrated its performance from three aspects. Our method showed better performance in distinguishing different drivers of land degradation than the typical RESTREND. In addition, we also applied the RESTREND and P-RESTREND in the Songnen grasslands to detect the degradation situation. We found that the RESTREND missed some land degradation areas that could be detected by P-RESTREND. The results indicated that our method is more effective in large areas.

## Figures and Tables

**Table 1:** VPR of the three trials at the overall level.

Trials	Type of Method	NDVI Period	Precipitation Period	R^2^
1	All study area	May–September	May–September	0.38
2	Pixel-by-pixel	Growing season	Growing season	0.35
3	Pixel-by-pixel	Growing season	Growing season and pre-growing season	0.45

**Table 2:** Percentage of pixels among the seven different categories detected by P-RESRTREND and RESTREND.

Method	LR1	LR2	LR3	LD1	LD2	LD3	NSC
P-RESREND	8.30	11.26	7.81	0.34	1.08	1.02	70.19
RESTREND	6.43	12.52	6.73	0.26	0.87	0.76	72.43
